# Lung injury induced by short-term mechanical ventilation with hyperoxia and its mitigation by deferoxamine in rats

**DOI:** 10.1186/s12871-020-01089-5

**Published:** 2020-08-01

**Authors:** Xiao-Xia Wang, Xiao-Lan Sha, Yu-Lan Li, Chun-Lan Li, Su-Heng Chen, Jing-Jing Wang, Zhengyuan Xia

**Affiliations:** 1 Department of Anesthesiology, First Hospital of Lanzhou University, Lanzhou, 730000 People’s Republic of China; 2 Department of Anesthesiology, The University of Hong Kong, Hong Kong, 999077 People’s Republic of China; 3 Department of Anesthesiology, Affiliated Hospital of Guangdong Medical University, Zhanjiang, 524000 People’s Republic of China

**Keywords:** Hyperoxia acute lung injury, Mechanical ventilation, Deferoxamine, Lung surfactant protein, Xanthine oxidase, Glutathion reductase

## Abstract

**Background:**

Long-term mechanical ventilation with hyperoxia can induce lung injury. General anesthesia is associated with a very high incidence of hyperoxaemia, despite it usually lasts for a relatively short period of time. It remains unclear whether short-term mechanical ventilation with hyperoxia has an adverse impact on or cause injury to the lungs. The present study aimed to assess whether short-term mechanical ventilation with hyperoxia may cause lung injury in rats and whether deferoxamine (DFO), a ferrous ion chelator, could mitigate such injury to the lungs and explore the possible mechanism.

**Methods:**

Twenty-four SD rats were randomly divided into 3 groups (*n* = 8/group): mechanical ventilated with normoxia group (MV group, FiO_2_ = 21%), with hyperoxia group (HMV group, FiO_2_ = 90%), or with hyperoxia + DFO group (HMV + DFO group, FiO_2_ = 90%). Mechanical ventilation under different oxygen concentrations was given for 4 h, and ECG was monitored. The HMV + DFO group received continuous intravenous infusion of DFO at 50 mg•kg^− 1^•h^− 1^, while the MV and HMV groups received an equal volume of normal saline. Carotid artery cannulation was carried out to monitor the blood gas parameters under mechanical ventilation for 2 and 4 h, respectively, and the PaO_2_/FiO_2_ ratio was calculated. After 4 h ventilation, the right anterior lobe of the lung and bronchoalveolar lavage fluid from the right lung was sampled for pathological and biochemical assays.

**Results:**

PaO_2_ in the HMV and HMV + DFO groups were significantly higher, but the PaO_2_/FiO_2_ ratio were significantly lower than those of the MV group (all *p* < 0.01), while PaO_2_ and PaO_2_/FiO_2_ ratio between HMV + DFO and HMV groups did not differ significantly. The lung pathological scores and the wet-to-dry weight ratio (W/D) in the HMV and HMV + DFO groups were significantly higher than those of the MV group, but the lung pathological score and the W/D ratio were reduced by DFO (*p* < 0.05, HMV + DFO vs. HMV). Biochemically, HMV resulted in significant reductions in Surfactant protein C (SP-C), Surfactant protein D (SP-D), and Glutathion reductase (GR) levels and elevation of xanthine oxidase (XOD) in both the Bronchoalveolar lavage fluid and the lung tissue homogenate, and all these changes were prevented or significantly reverted by DFO.

**Conclusions:**

Mechanical ventilation with hyperoxia for 4 h induced oxidative injury of the lungs, accompanied by a dramatic reduction in the concentrations of SP-C and SP-D. DFO could mitigate such injury by lowering XOD activity and elevating GR activity.

## Background

During the course of general anesthesia, inhalation of high fraction of inspired oxygen (FiO_2_) is usually used to prevent hypoxaemia in emergencies and to enhance patients’ tolerance to apnea and hypopnea [1]. However, excessively high concentration of oxygen supplied during the surgery may sometimes lead to hyperoxaemia [2, 3]. A multi-center clinical study showed that the incidence of hyperoxaemia during general anesthesia reaches up to 83% [4]. Although the effect of hyperoxia in critical illness is still inconclusive [5], and the risk of hyperoxaemia in craniocerebral trauma or stroke was also ambiguous,observational studies showed a close relationship between hyperoxaemia and increased mortality in critically ill patients [6, 7], and it can also lead to poor prognosis in patients with hypoxic-ischemic encephalopathy [8]. Besides, as shown in the animal experiments, long-term exposure to the hyperoxic environment caused oxidative injury of the lungs [9], and another clinical study indicated that long-term hyperoxia increased the risks of lung complications in humans, including pneumonia, atelectasis and pulmonary edema [10]. However, it remains unclear whether or not short-term hyperoxia also exerts an adverse impact on the lung tissues. Since most of the surgeries under general anesthesia are accomplished over relatively a short time, this study was concerned whether mechanical ventilation with hyperoxia for 4 h would cause oxidative injury of the lungs.

Pulmonary surfactant (PS) is a lipoprotein secreted by alveolar epithelial type II cells (AECII), and its main bioactive components are surfactant proteins (SPs), including SP-A, SP-B, SP-C and SP-D. Among them, SP-C is a hydrophobic polypeptide derived from AECII and involved in the adjustment of alveolar surface tension. SP-C-deficient mice are found to be susceptible to bacterial and viral infections [11, 12]. SP-D regulates the immune and inflammatory responses and serves as a marker for alveolar integrity. Changes in SP-D content are positively correlated to the severity of lung injury [13, 14]. Experiments have shown [15] that long-term exposure (t > 24 h) to atmospheric oxygen concentration above 90% will lead to dynamic changes of SP. At present, there have been no relevant reports as to the potential influence of short-term mechanical ventilation with hyperoxia on SP.

Deferoxamine (DFO) is a ferrous ion chelator, which is currently used to treat the diseases caused by iron overload, for example, acute iron poisoning and chronic iron allergy. Animal studies have shown that [16, 17] DFO can alleviate the oxidative stress induced by reactive oxygen species (ROS) in rat pulmonary contusion, which is further related to an increase in the activity of xanthine oxidase (XOD). In addition, DFO can also increase the content of glutathione (GSH), clearing excessive ROS and reducing the injury done by ROS to the cells [18]. Britt et al. reported [19, 20] that the regulation of GSH level had a protective effect against the hyperoxia-induced lung injury. Glutathion reductase (GR) is a key enzyme regulating the GSH level and helping protect the cells from the oxidative stress injury. In the present, we aimed to clarify whether DFO had a protective effect against the lung injury caused by mechanical ventilation with hyperoxia and whether DFO worked by influencing the activities of XOD and GR.

Mechanical ventilation with hyperoxia was implemented to the rats for 4 h. Then we discussed whether short-term hyperoxia could induce the oxidative stress injury of the lungs or the associated changes in SP. Furthermore, continuous infusion of DFO was performed during mechanical ventilation so as to verify whether DFO had a protective effect against the lung injury induced by mechanical ventilation with hyperoxia.

## Methods

### Section of animals

Twenty-four healthy adult male SD rats, each weighing 200 ± 10 g on average, were provided by the Laboratory Animal Center of Lanzhou University School of Medicine. Before the formal experiment began, the rats were acclimatized for 1 week in a quiet environment, with natural illumination, temperature 20–26 °C, diurnal range of temperature ≤ 4 °C, and humidity 40–60%. The experimental design conformed to the ethical standards for animal experiments at the First Hospital of Lanzhou University.

### Animal model and treatment

Using a random number table, the rats were divided into 3 groups, with 8 rats in each group, namely, mechanical ventilation with normoxia group (MV group), mechanical ventilation with hyperoxia group (HMV group) and mechanical ventilation with hyperoxia+DFO group (HMV + DFO group). Anesthesia was induced by intraperitoneal injection of 2% Phenobarbital sodium (0.2 ml/100 g). The rats were immobilized to the operating table in a supine position. Heart rate (HR) was monitored. Tail vein puncture and cannulation were performed to prepare for the transfusion. The neck was fully exposed. The left carotid artery was punctured and cannulated under a sterile condition. Posterior to the exposed trachea a T-shaped incision about 2–3 mm long was made and the endotracheal tube was inserted and connected to the ventilator for small animals (HX-100E, Chengdu, China) for mechanical ventilation. The respiratory parameters were configured [21]: tidal volume 10 ml/kg, frequency 40–60 times/min, and inspiration-to-expiration ratio 1:1. The MV group received mechanical ventilation with 21% oxygen in air. The HMV and HMV + DFO groups received mechanical ventilation with 90% oxygen concentration, for 4 h continuously.

During mechanical ventilation, rats in the HMV + DFO group received continuous infusion of DFO via the tail vein (50 mg•kg^− 1^•h^− 1^, Novartis, Shanghai, China) for 4 h. The MV and HMV groups were given an equal volume of normal saline (1 ml/h). At 2 and 4 h, 0.2 ml of blood was drawn from the carotid artery for blood gas analysis. The respiratory rate was adjusted based on the results of blood gas analysis to maintain PaCO_2_ at 35–45 mmHg. Anesthetic maintenance was achieved by intermittent intraperitoneal injection of 2% phenobarbital sodium and using fentanyl (12 μg/kg) according to the changes in HR during the ventilation. At the completion of the experiments, the rats were euthanized with over dose of phenobarbital sodium injection.

### Blood gas analysis

At 2 and 4 h of mechanical ventilation, blood samples were collected from the carotid artery for blood gas analysis. PH, PaCO_2_ and PaO_2_ were recorded, and PaO_2_/FiO_2_ ratio was calculated.

### Lung wet/dry ratio (W/D)

After mechanical ventilation for 4 h, the rats were euthanized. The chest was opened, and the right posterior lobe of the lung was harvested. The dry weight (W) of the lung tissue was determined using a precision electronic balance. Then the lung tissues were immediately placed into a drying oven for constant temperature drying at 80 °C for 72 h. After that, the lung tissues were weighed again until constant weight, which was the dry weight (D). The wet/dry weight ratio was calculated by (W/D) = W (g)/D (g) × 100%, and its changes were monitored.

### Histological evaluation

The right anterior lobe of the lung was harvested and fixed inflated, and prepared into slices 4 μm thick. HE staining was performed, and histological changes were observed under the optical microscope. Pathological scoring was performed by a pathologist who was blinded with the group assignment or experiment design. The scoring criteria [22] was as follows: 0 point, normal alveolar structure, mesenchyme and pulmonary vessels; 1 point, mild damage of the alveolar structure, small amount of inflammatory cells in the mesenchyme, and the scope of bleeding and edema in the mesenchyme and alveolar spaces less than 25%; 2 points: moderate damage of the alveolar structure, a large amount of inflammatory cells in the mesenchyme and some alveolar spaces, widened mesenchyme, congestion in the capillaries, and scope of bleeding and edema in the alveolar spaces 25–50%; 3 points: severe damage of the alveolar structure, agglomeration of inflammatory cells in most alveoli and mesenchyme, apparently widened mesenchyme, and the scope of bleeding and edema in the alveolar spaces 50–75%.

### Assessment of SP-C, SP-D, XOD and GR

#### Tissue preparation

The upper end of the trachea and right hilum were ligated. The sterile endotracheal tube was replaced and connected to a 5 ml needle. Next, 2.5 ml of pre-cooled phosphate-buffered saline (PBS) was injected into the needle for left alveolar lavage. After two aspirations, the lavage fluid was drawn into a centrifuge tube. The lavage was repeated for 3 times, and it was considered successful if the recovery rate was above 80% [23]. The collected bronchoalveolar lavage fluid (BALF) was centrifuged at 3000 r/min at 4 °C for 10 min, and the supernatant was collected. Meanwhile, 110 mg of right middle lobe of the lung was harvested and washed with PBS previously preserved at 4 °C. Impurities were removed from the lung tissues. The lung tissues were weighed and added with PBS 9 times the mass of the lung tissues. Lung tissue homogenate was prepared in an ice-water bath using a homogenizer and centrifuged at 3000 r/min at 4 °C for 15 min. The supernatant was collected.

#### Detection of SP-C, SP-D, XOD and GR in BALF and lung tissue homogenate

Enzyme Linked ImmunoSorbent Assay (ELISA) was performed to detect the concentrations of SP-C, SP-D, XOD and GR in BALF and lung tissue homogenate. All detection procedures were undertaken according to the instruction manual of the ELISA kits (Mlbio, Shanghai, China) for SP-C (sensitivity, <0.1 pg/ml), SP-D (sensitivity, < 0.1 pg/ml), XOD (sensitivity, < 0.1 U/L) and GR (sensitivity, < 1.0 mIU/ml) in rats.

### Statistical analysis

All statistical analyses were performed using SPSS 22.0 software. It was verified by the Shapiro-Wilks normality test that all original data obeyed a normal distribution. Next, Bartlett’s test was used to determine whether the independent samples satisfied homogeneity of variances. The pathological scores of lung tissues and the data on W/D ratio and concentrations of SP-C, SP-D and GR satisfied homogeneity of variances. One-way ANOVA was performed to compare three groups of data. SNK test was performed for multiple comparisons. Data on XOD concentration did not satisfy homogeneity of variances, and so Tamhane’s T2 was performed. The results of arterial blood gas analysis were compared by the repeated measures ANOVA. Data were expressed as mean ± standard deviation (^−^x ± s). *P* < 0.05 was taken to indicate significant difference.

## Results

### Data of blood gases

At 2 and 4 h of mechanical ventilation, blood gas analysis showed that as compared with the MV group, PaO_2_ increased in the HMV and HMV + DFO groups (*P<*0.001), while PaO_2_/FiO_2_ ratio decreased (*P<*0.001). There were no significant differences in PaO_2_ and PaO_2_/FiO_2_ ratio between the HMV and HMV + DFO groups (*P*>0.05). At 2 and 4 h, there were no significant differences in PH and PaCO_2_ between the three groups (*P* > 0.05) (Table 1).

**Table 1:** Arterial blood gas analysis results at 2 and 4 h (mean ± SD)

Groups	Time (h)	PaO_**2**_ (mmHg)	PaCO_**2**_ (mmHg)	PH	PaO_**2**_/FiO_**2**_ (mmHg)
**MV**	2	89.25 ± 3.85	34.13 ± 3.76	7.35 ± 0.05	425.00 ± 18.31
	4	90.25 ± 4.53	37.13 ± 3.18	7.38 ± 0.04	429.76 ± 21.56
**HMV**	2	204.88 ± 7.57*	33.38 ± 5.01	7.33 ± 0.05	227.64 ± 8.41*
	4	208.75 ± 7.44*	36.63 ± 3.38	7.35 ± 0.03	231.94 ± 8.26*
**HMV + DFO**	2	205.50 ± 9.64*	36.50 ± 4.07	7.34 ± 0.04	228.33 ± 10.70*
	4	208.00 ± 6.95*	37.88 ± 4.88	7.36 ± 0.04	231.11 ± 7.72*

Table 1 Data of blood gases. Values are displayed as means ± SD

*Group MV* Mechanical ventilation with normoxia group, *Group HMV* Mechanical ventilation with hyperoxia group, *Group HMV + DFO* Mechanical ventilation with hyperoxia+DFO group

*P = 0.000 as compared with the MV group

### Lung tissue observation results and pathology score

**Fig. 1:**
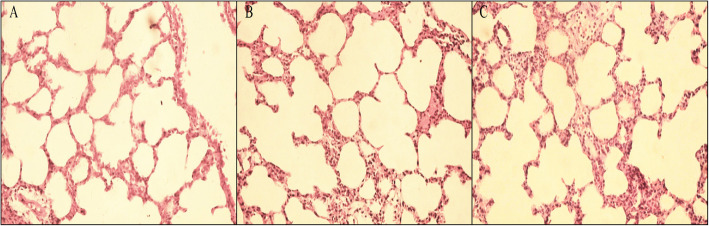
Lung tissue observation results. **a** shows the pathological changes of lung tissues in the MV group (mechanical ventilation with normoxia group); **b** shows the pathological changes of lung tissues in the HMV group (mechanical ventilation with hyperoxia group); **c** shows the pathological changes of lung tissues in the HMV + DFO group (mechanical ventilation with hyperoxia group+DFO). As compared with the HMV group, the lung injury was greatly alleviated

**Fig. 2:**
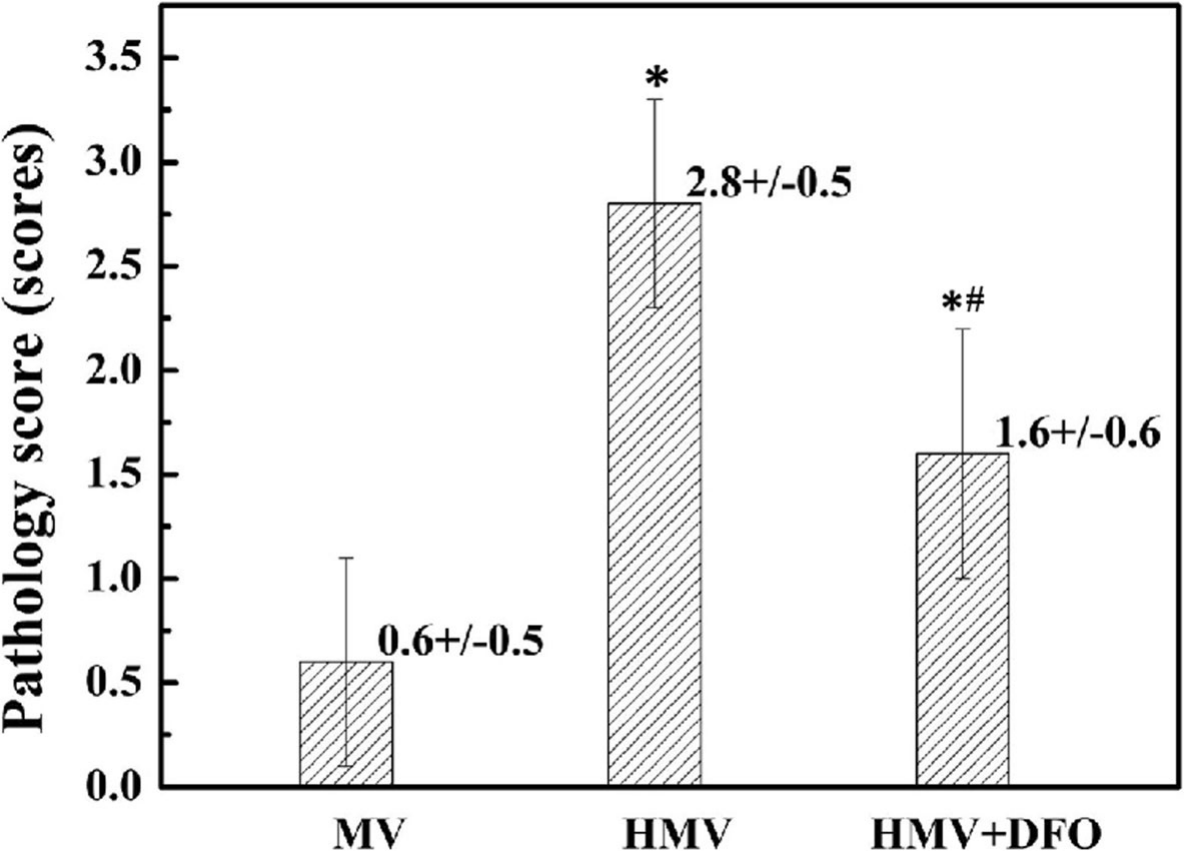
Lung tissue pathology score. Values are displayed as means ± SD. Group MV: Mechanical ventilation with normoxia group; Group HMV: Mechanical ventilation with hyperoxia group; Group HMV + DFO: Mechanical ventilation with hyperoxia+DFO group. After mechanical ventilation with hyperoxia for 4 h, the pathological score of the lung tissues increased significantly as compared with the MV group (*P* = 0.000). After DFO treatment, the pathological score of the lung tissues decreased significantly as compared with the HMV group (*P* = 0.001), and it increased significantly as compared with the MV group (*P* = 0.015)

As to the pathological changes of the lung tissues, the alveolar structure was distinctively visualized and was basically intact in the MV group. There were few inflammatory cells in the pulmonary mesenchyme and alveolar spaces; the alveolar septum was not widened, and neither was there apparent dilation of the pulmonary vessels. In the HMV group, some of the alveolar walls fractured, with mild alveolar fusion. This mainly presented with widening of alveolar septa and congestion and dilation of the pulmonary vessels. There was exudation of inflammatory cells and fluid from the pulmonary mesenchyme and alveolar spaces. Abnormalities in the alveolar structure, morphology and size and the degree of exudation of inflammatory cells in the HMV + DFO group were significantly alleviated as compared with HMV group (Fig. 1). As compared with the MV group, the pathological scores of the lung tissues in the HMV and HMV + DFO groups increased significantly (*P<*0.001, *P<0.05*). As compared with the HMV group, the pathological scores of the lung tissues in the HMV + DFO group decreased significantly (*P<0.005*) (Fig. 2).

### Lung W/D ratio

**Fig. 3:**
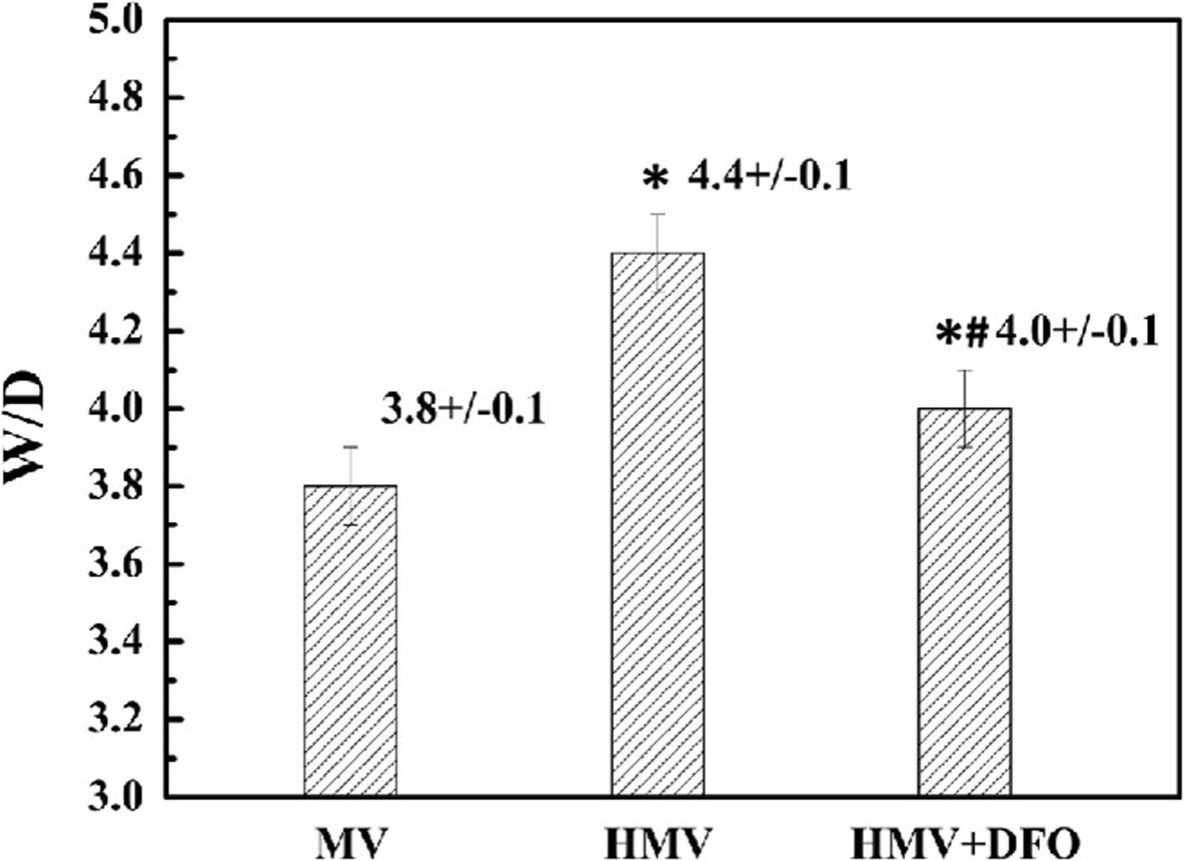
Lung W/D ratio. Values are displayed as means ± SD. Group MV: Mechanical ventilation with normoxia group; Group HMV: Mechanical ventilation with hyperoxia group; Group HMV + DFO: Mechanical ventilation with hyperoxia+DFO group. After mechanical ventilation with hyperoxia for 4 h, the W/D ratio of the lung tissues increased significantly as compared with the MV group (*P* = 0.000). After DFO treatment, the W/D ratio of the lung tissues decreased significantly as compared with the HMV group (*P* = 0.000), and it increased significantly as compared with the MV group (*P* = 0.000)

As compared with the MV group, the W/D ratio of the lung tissues in the HMV and HMV + DFO groups increased significantly (*P<*0.001), while the W/D ratio of the lung tissues in the HMV + DFO group decreased significantly (*P<*0.001) (Fig. 3).

### Changes of SP-C, SP-D, XOD and GR levels in BALF

**Fig. 4:**
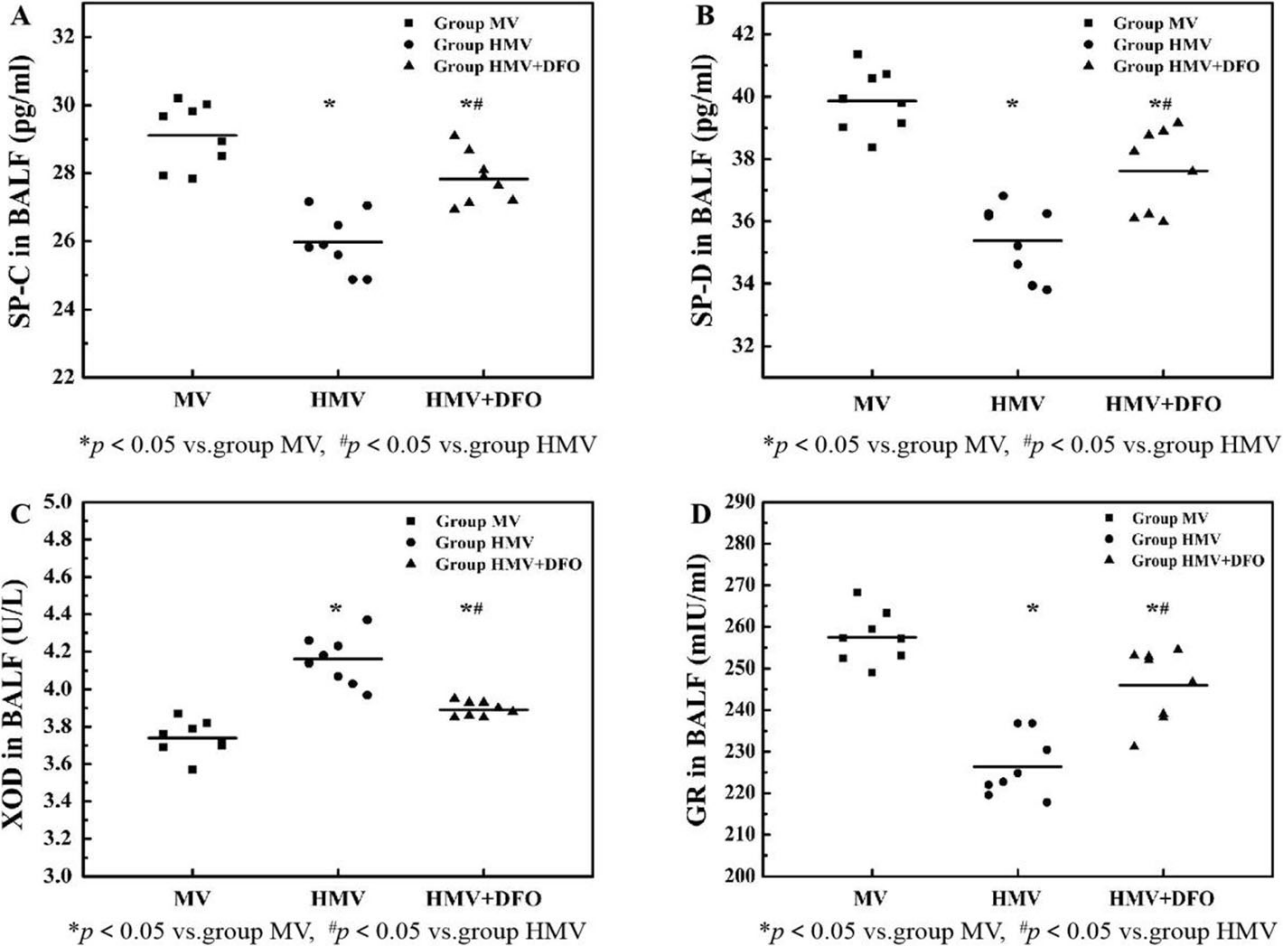
Changes of SP-C,SP-D,XOD and GR levels in BALF. Values are displayed as means ± SD. Group MV: Mechanical ventilation with normoxia group; Group HMV: Mechanical ventilation with hyperoxia group; Group HMV + DFO: Mechanical ventilation with hyperoxia+DFO group; **a** shows the comparison of SP-C concentration in BALF between the three groups; **b** shows the comparison of SP-D concentration in BALF between the three groups; **c** shows the comparison of XOD concentration in BALF; **d** shows the comparison of GR concentration in BALF

As compared with the MV group, the concentrations of SP-C, SP-D and GR in the BALF of the HMV and HMV + DFO group decreased significantly (*P<*0.001), while the XOD concentration increased significantly (*P<*0.001). As compared with the HMV group, the concentrations of SP-C, SP-D and GR in the BALF increased significantly in the HMV + DFO group (*P<*0.001), while the XOD concentration decreased significantly (*P<*0.001) (Fig. 4).

### Changes of SP-C, SP-D, XOD and GR levels in lung tissue homogenate

**Fig. 5:**
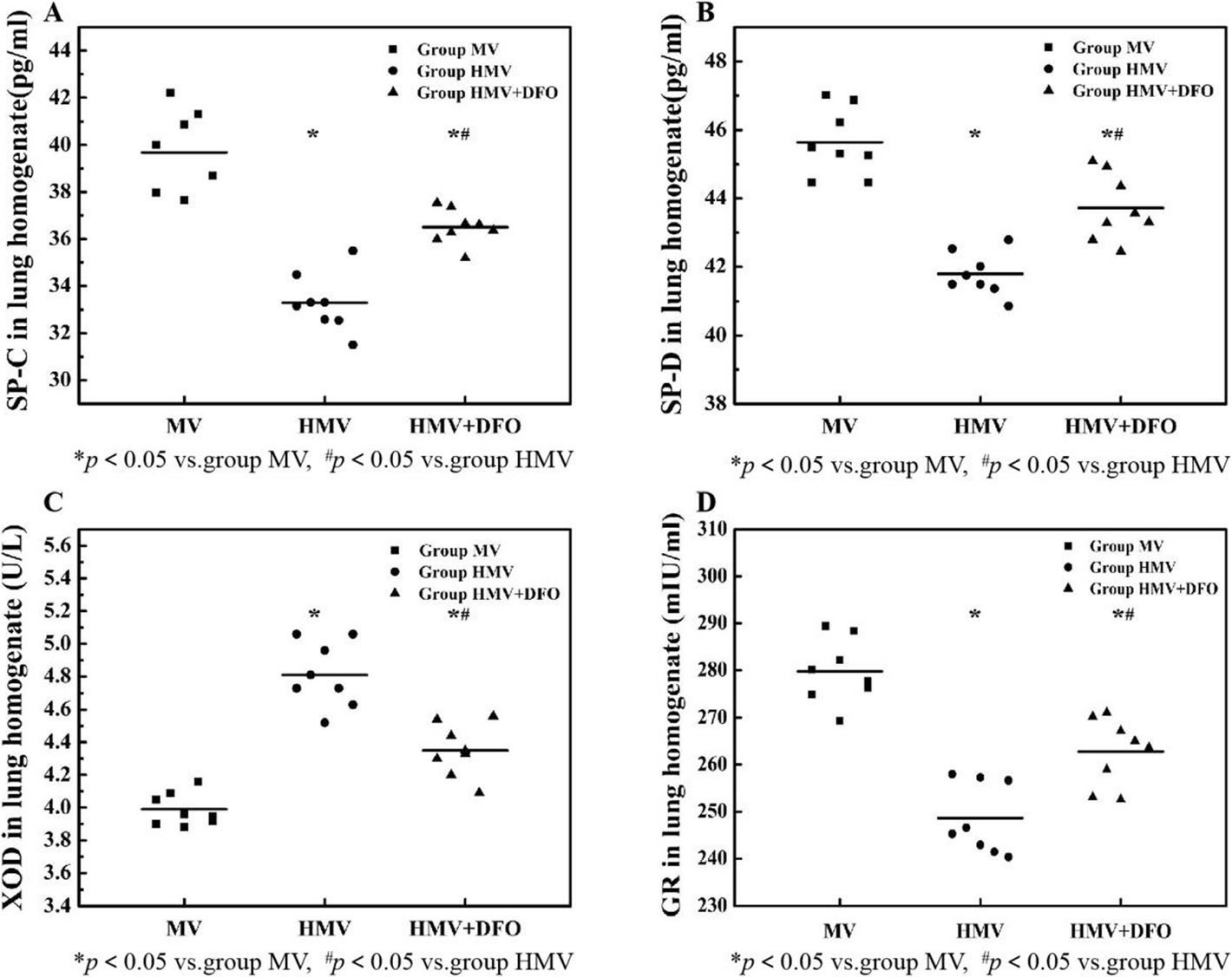
Changes of SP-C,SP-D,XOD and GR levels in lung tissue homogenate.Values are displayed as means ± SD. Group MV: Mechanical ventilation with normoxia group; Group HMV: Mechanical ventilation with hyperoxia group; Group HMV + DFO: Mechanical ventilation with hyperoxia+DFO group; **a** shows the comparison of SP-C concentration in the lung tissue homogenate between the three groups; **b** shows the comparison of SP-D concentration in the lung tissue homogenate between the three groups; **c** shows the comparison of XOD concentration in the lung tissue homogenate; **d** shows the comparison of GR concentration in the lung tissue homogenate

As compared with the MV group, the concentrations of SP-C, SP-D and GR in the lung tissue homogenate of the HMV and HMV + DFO group decreased significantly (*P<*0.001), while the XOD concentration increased significantly (*P<*0.001). As compared with the HMV group, the concentrations of SP-C, SP-D and GR in the lung tissue homogenate increased significantly in the HMV + DFO group (*P<*0.001), while the XOD concentration decreased significantly (*P<*0.001). (Fig. 5).

## Discussion

In the present study, lung tissue pathology and the increase of lung tissue W/D ratio suggested that mechanical ventilation with hyperoxia for 4 h caused lung injury. The arterial blood gas analysis results obtained at 2 and 4 h of ventilation showed that the rats were in a state of hyperoxemia evidenced as elevated PaO_2_.The decreases in the concentrations of SP-C, SP-D and GR in the BALF and lung tissue homogenate along with a considerable increase in the XOD concentration were evidences that hyperoxia affected the secretions of SP-C, SP-D, GR and XOD. After DFO treatment, the lung injury of rats was alleviated, while concentrations of SP-C, SP-D and GR increased as compared to those under hyperoxia without DFO treatment, accompanied with a reduction of the increased XOD. This indicated that DFO had a protective effect against the lung injury caused by short-term hyperoxia, and its working mechanism might be related to an increase in the activity of GR and a decrease in the activity of XOD.

Long-term exposure to a hyperoxic environment may lead to lung injury [9, 24]. Kawamura showed that continuous exposure of rats to 98% atmospheric oxygen for 60 h would cause oxidative injury of the lungs. Another meta-analysis pointed out that critically ill patients after mechanical ventilation with hyperoxia usually had poor outcome associated with the lungs [25, 26]. However, whether short-term mechanical ventilation with hyperoxia will cause lung injury remains a topic not getting enough attention. Here, PaO_2_/FiO_2_ ratio, W/D ratio and pathological scores of the lung tissues at 2 and 4 h of mechanical ventilation with hyperoxia were much higher than those of the control group. Ferguson reported that PaO_2_/FiO_2_ ratio was an accurate indicator of the oxygenation status of the organism under oxygen inhalation. A PaO_2_/FiO_2_ ratio below 300 mmHg usually indicates respiratory insufficiency [27]. It should be noted that the relation between PaO_2_/FiO_2_ ratio and FiO_2_ is nonlinear, factors that influence PaO_2_/FiO_2_ ratio are not only arise from the change of FiO_2_, but also from the effect of intrapulmonary shunt affected by FiO_2_ [28]. Therefore, using PaO_2_/FiO_2_ alone to evaluate lung injury has certain limitations in our experiment. It should be noted that high FiO_2_ may cause absorptive atelectasis, and the atelectasis formation would possibly cause the oxygenation impairment. W/D ratio is an objective indicator of water content of the lung tissues. If it is above 4, pulmonary edema is usually indicated; and the higher the ratio, the more severity the pulmonary edema. In the present study, pulmonary edema was observed after mechanical ventilation with hyperoxia for 4 h, which was accompanied by an increase in the pathological score of the lung tissues. This further indicated that short-term mechanical ventilation with hyperoxia would induce injury of the lung tissues in rats. After DFO treatment, the lung injury was greatly alleviated, while the PaO_2_/FiO_2_ ratio did not improve substantially. This may be related to the fact that the intrapulmonary arteriovenous shunting caused by pulmonary edema affected PaO_2_.

ROS generated by hyperoxia and the resulting excessive oxidative stress are important working mechanisms for lung injury [29, 30]. Early research indicated that pulmonary vascular endothelial cells are more sensitive to high concentrations of oxygen, and damage to pulmonary vascular endothelial cells is an important cause of death in rats exposed to 100% O_2_ for a prolonged time [31]. Recent studies [32, 33] have shown that AEC II was the primary target cells for ROS. When the lung tissues are in an oxidative stress status, a large amount of ROS is released into the alveolar spaces, inducing the apoptosis of AECII, which is further related to the hyperoxia-induced lung injury. In case of hyperoxia-induced lung injury, AECII is injured, which further influences the secretion of PS. PS, composed of 10% SP and 90% phospholipid approximately, fulfills the functions of reducing alveolar surface tension, maintaining alveolar stability, inhibiting inflammatory response and enhancing the phagocytic function of alveolar macrophages. When the content of SP changes, it may cause pulmonary insufficiency and atelectasis [34]. SP-C is a hydrophobic glycoprotein secreted by AECII, which promotes the absorption of phospholipid and its distribution to the air-liquid interface of the lung. This will further reduce alveolar surface tension and ensure alveolar integrity and its normal biological role [35]. Sano et al. showed that SP-D knockout mice suffered from alveolar structural abnormalities, metabolic impairment of PS and host defense deficiency. SP-D deficiency could cause pulmonary injury to a certain degree [36]. In the present study, after mechanical ventilation with hyperoxia for 4 h, inhalation of hyperoxic gases led to alveolar injury of rats. It was thus inferred that the AECII structure was damaged. As the release of SP-C and SP-D from AECII was inhibited, the concentrations of SP-C and SP-D in the lung tissue homogenate and BALF decreased significantly. Moreover, as the concentrations of SP-C and SP-D changed, the composition of PS in AECII was altered. As a result, PS failed to perform the functions of reducing alveolar surface tension and maintaining alveolar volume, which further induced structural and functional abnormalities of the lung tissues. Thus the vicious cycle began, aggravating pulmonary edema and promoting lung injury. A significant increase in the concentrations of SP-C and SP-D was noted after DFO treatment, indicating an alleviation of pulmonary edema and lung injury and also a protective effect of DFO against the lung injury caused by hyperoxia.

XOD and GR are key enzymes in the oxidant-antioxidant system [37, 38]. Under normal conditions, XOD is inactive, and its activity increases when tissues are in an oxidative stress status. The active form of XOD can catalyze oxidation of xanthine, producing a large amount of ROS and mediating the peroxidation tissue injury. In the oxidative response, GR can clear excessive ROS by maintaining the content of reduced GSH, thus alleviating peroxidation tissue injury. Therefore, an increase in the activity of XOD and a decrease in the activity of GR usually suggest an increase in the ROS level and hence intense oxidative stress. In the present study, the XOD concentration increased and GR concentration decreased after mechanical ventilation with hyperoxia. This indicated ROS production-clearing imbalance and disorder of the oxidant-antioxidant enzyme system after mechanical ventilation with hyperoxia for 4 h, which further contributed to the lung injury.

DFO has proven to inhibit lipid peroxidation by reducing ROS generation [39]. Hybertson et al. found through animal experiments [17, 40] that DFO cleared excessive ROS by inhibiting XOD activity, reducing ROS production and increasing GSH content, which finally alleviated oxidative injury of the cells. GSH can protect lung tissues from hyperoxia-induced injury and GSH level reflects the activity of GR to a certain extent. In response to oxidative stress, GR activity is enhanced, and the content of reduced GSH increases as well, thus propelling ROS clearing and exerting an antioxidant effect. We found that (1) after DFO treatment, the XOD concentration decreased dramatically, suggesting that DFO alleviated the hyperoxia-induced lung injury by inhibiting XOD activity; (2) a significant increase in GR concentration after DFO treatment indicated that DFO might exert a protective effect for the lungs by enhancing GR activity and increasing the content of reduced GSH; (3) given a generally low ROS level, the injury to AECII was mild, and the reduction in the concentrations of SP-C and SP-D was redressed significantly by DFO, which finally alleviated pulmonary edema.

In the course of the experiment, the lack of positive end-expiratory pressure (PEEP) or lung recruitment maneuver applied and other related protective lung ventilation strategies for mechanically ventilated rats may affect our observations of lung tissue damage, which is one of the limitations of our experiment. In further researches, it is necessary to solve the above problems and take relevant measures (such as the application of PEEP to avoid or prevent the occurrence of atelectasis, thereby reducing the deviation of the experimental results.

## Conclusions

Taken together, mechanical ventilation with hyperoxia for 4 h caused oxidative injury and a dramatic reduction in the concentrations of SP-C and SP-D in the lung tissue homogenate and BALF. This further led to respiratory impairment and pulmonary edema. DFO could alleviate the lung injury induced by mechanical ventilation with hyperoxia, exerting a protective effect for the lungs. Its working mechanism might be related to a reduction in XOD activity, an increase in the SP-C concentration and GR activity and alleviation of injury to AECII.

## Data Availability

The datasets used and/or analyzed during the current study available from the corresponding author on reasonable request.

## Abbreviations

FiO_2_: Fraction of inspired oxygen
PS: Pulmonary Surfactant
AECII: Alveolar epithelial type II cells
SPs: Surfactant Proteins
DFO: Deferoxamine
ROS: Reactive Oxygen Species
XOD: Xanthine Oxidase
GSH: Glutathione
GR: Glutathion Reductase
HR: Heart Rate
PBS: Phosphate-buffered Saline
BALF: Bronchoalveolar Lavage Fluid
ELISA: Enzyme Linked ImmunoSorbent Assay
PEEP: Positive end-expiratory pressure
